# Crystal structure of the BoNT/A2 receptor-binding domain in complex with the luminal domain of its neuronal receptor SV2C

**DOI:** 10.1038/srep43588

**Published:** 2017-03-02

**Authors:** Roger M. Benoit, Martin A. Schärer, Mara M. Wieser, Xiaodan Li, Daniel Frey, Richard A. Kammerer

**Affiliations:** 1 Laboratory of Biomolecular Research, Division of Biology and Chemistry, Paul Scherrer Institute, CH-5232 Villigen PSI, Switzerland

## Abstract

A detailed molecular understanding of botulinum neurotoxin (BoNT)/host-cell-receptor interactions is fundamental both for developing strategies against botulism and for generating improved BoNT variants for medical applications. The X-ray crystal structure of the receptor-binding domain (H_C_) of BoNT/A1 in complex with the luminal domain (LD) of its neuronal receptor SV2C revealed only few specific side-chain – side-chain interactions that are important for binding. Notably, two BoNT/A1 residues, Arg 1156 and Arg 1294, that are crucial for the interaction with SV2, are not conserved among subtypes. Because it has been suggested that differential receptor binding of subtypes might explain their differences in biological activity, we determined the crystal structure of BoNT/A2-H_C_ in complex with SV2C-LD. Although only few side-chain interactions are conserved between the two BoNT/A subtypes, the overall binding mode of subtypes A1 and A2 is virtually identical. In the BoNT/A2-H_C_ – SV2C complex structure, a missing cation-π stacking is compensated for by an additional salt bridge and an anion-π stacking interaction, which explains why the binding of BoNT/A subtypes to SV2C tolerates variable side chains. These findings suggest that motif extensions and a shallow binding cleft in BoNT/A-H_C_ contribute to binding specificity.

Botulinum neurotoxins (BoNTs) comprise a family of seven serologically distinct neurotoxin types, designated BoNT/A through BoNT/G, that cause botulism, a rare but potentially fatal paralytic disease, in vertebrates^1^,^2^. Human botulism is caused by BoNTs A, B, E but only rarely F. Serotypes C and D cause illness in other species, such as mammals, birds and fish. No cases of botulism have been reported for BoNT/G^1^,^3^. BoNTs are produced by some gram-positive bacteria of the genus *Clostridium*, but a non-Clostridial BoNT-like protein has also been recently identified in *Weissella oryzae* SG25T^4^. Moreover, BoNT variety is further increased by the existence of different subtypes and mosaic proteins^1^,^5^. For BoNT/A, eight different subtypes have been identified^1^,^6^, some of which differ in their intoxication properties^7^,^8^.

All BoNTs share the same domain organisation^1^,^2^,^6^. BoNT molecules are synthesized as single polypeptide chains that are processed into mature toxins by cleavage into two subunits, a 50 kDa light chain (LC) and a 100 kDa heavy chain (HC), that remain connected through a disulfide bond.

The LC is a zinc endopeptidase that specifically cleaves a member of the soluble N-ethylmaleimide-sensitive-factor attachment receptor (SNARE) family of proteins that are key components of the vesicular fusion machinery within presynaptic nerve terminals^1^,^2^. BoNTs A, C and E cleave synaptosomal-associated protein 25 (SNAP-25); BoNTs B, D, F, and G cut vesicle-associated membrane protein (VAMP), and BoNT/C cleaves syntaxin. SNARE cleavage results in the blockade of acetylcholine release at the neuromuscular junction, which causes long-lasting flaccid paralysis of muscles.

The HC consists of a 50 kDa N-terminal translocation domain (H_N_) and a C-terminal receptor-binding domain (H_C_) of similar size^1^,^2^,^6^. A dual interaction mode involving different receptors appears to be important for neuro-specific binding of BoNTs^9^. All serotypes share a binding site for the interaction with the oligosaccharide portion of a polysialoganglioside^6^. A second, non-conserved binding site that binds different receptors has been identified in several BoNTs. BoNT/A, BoNT/D, BoNT/E and BoNT/F bind synaptic vesicle protein 2 (SV2)^1^,^6^. BoNT/B and BoNT/G recognize a short segment of the luminal domain of the synaptic vesicle protein synaptotagmin^1^,^6^. A second receptor for BoNT/C has so far not been identified.

Upon binding to neuronal cells, BoNTs undergo receptor-mediated endocytosis. The acidic environment in the endosome is believed to trigger a conformational change in H_N_, resulting in the formation of a transmembrane channel through which the protease is translocated across the synaptic vesicle membrane^1^. Once inside the reducing environment of the cytosol, the disulfide bond is reduced and the protease is released^10^.

We recently determined the crystal structure of the BoNT/A1-H_C_ in complex with the luminal domain (LD) of its human protein receptor SV2C^6^,^11^,^12^. BoNT/A1-H_C_ uses an exposed open β-strand to bind to the outermost, unprotected, open β-strands of SV2C-LD. Although the toxin-receptor interaction is dominated by backbone-backbone hydrogen bonds, the importance for binding specificity of prominent side–chain–side–chain interactions seen in the structure was demonstrated by site-directed mutagenesis. Interestingly, these side chains are not conserved amongst BoNT/A subtypes^6^. Because these differences are particularly pronounced in BoNT/A2, we decided to investigate the interaction of this subtype with SV2C-LD in detail.

## Results and Discussion

### BoNT/A2-H_C_ strongly binds to human SV2C-LD despite important side-chain differences at the binding site compared to BoNT/A1- H_C_

**Figure 1:**
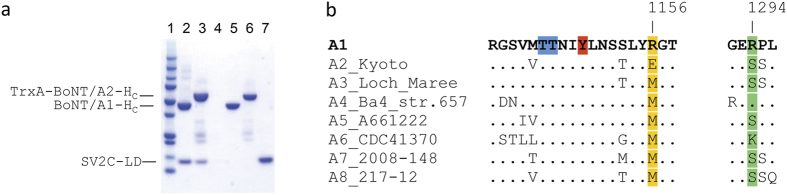
BoNT/A2-H_C_ strongly binds to human SV2C-LD. (**a**) Pulldown experiments using untagged SV2C-LD and His-tagged BoNT/A-H_C_ proteins. 1) Precision Plus Protein Standard (Bio-Rad); 2) 6xHis-BoNT/A1-H_C_ and SV2C-LD; 3) 6xHis-TrxA-BoNT/A2-H_C_ and SV2C-LD and 4) untagged SV2C-LD. 5–7) Purified proteins that were used for the pulldown assays: 5) 6xHis-BoNT/A1-H_C_; 6) 6xHis-TrxA-BoNT/A2-H_C_ and 7) untagged SV2C-LD. The additional bands in the A2 sample might represent degradation products or impurities that could not be removed during purification. (**b**) Alignment of BoNT/A subtypes. Residues 1156 and 1294, which differ between A1 and A2, are highlighted in yellow and green, respectively. Thr 1145, Thr 1146 (blue) and Tyr 1149 (red), three conserved residues that form identical interactions in all published BoNT/A-H_C_ - SV2C-LD complex structures, are highlighted in the A1 sequence. Residues that are identical to the A1 sequence are represented as dots.

It is currently not known how small amino-acid differences influence the biological activity of BoNT subtypes. It has been suggested that differential receptor binding of subtypes might explain the different toxin subtype characteristics^7^,^8^,^13^. Our previous work on the BoNT/A1-H_C_ - SV2C-LD complex supports this hypothesis. The crystal structure, as well as pulldown experiments and fluorescence anisotropy studies, demonstrated a pivotal role of several side-chain interactions for the binding of the toxin to its protein receptor^6^,^11^,^12^. Some of these residues are, however, not conserved between BoNT/A subtypes (Fig. 1) or between the SV2C-LD of different species^6^,^11^. In particular, disruption of the cation-π stacking interaction between Arg 1156 of BoNT/A1 and Phe 563 of human SV2C by mutagenesis strongly affected complex formation^12^. Mutation of Arg 1294 of BoNT/A1 to alanine also resulted in decreased binding to SV2C-LD. However, Arg 1294 is not well defined in one of the two copies of the complex in the asymmetric unit of the crystal and hence probably does not form ordered bonds in solution. Accordingly, we speculated that this residue contributes to the overall positive charge of the receptor-binding site in BoNT/A1-H_C_ and may therefore play an important role in early recognition events. BoNT subtype A2 contains a Glu at position 1156 and a Ser at position 1294 and can neither form a cation-π interaction, nor contribute to an overall positive net charge of the receptor binding domain. We were therefore surprised that human SV2C-LD nevertheless bound strongly to BoNT/A2-H_C_ in pulldown assays (Fig. 1). The result furthermore raised the question whether BoNT/A2 interacts with SV2C via a binding mode completely different from BoNT/A1. In this context, it is noteworthy that BoNT/B^14^ binds to its peptide receptor synaptotagmin-II in a completely different orientation than the mosaic toxin BoNT/DC^6^,^15^.

### BoNT/A1 and BoNT/A2 share the same overall SV2C-LD binding mode

**Figure 2:**
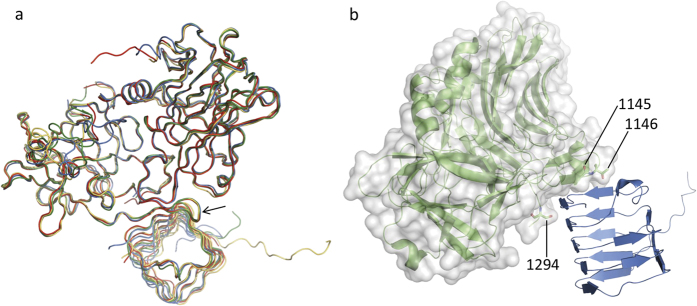
Superimposition of the crystal structures of the four known of BoNT/A-H_C_ – SV2C-LD complexes reveals an identical receptor-toxin binding mode. (**a**) PDB entry 5jmc (BoNT/A1-H_C_ – rat SV2C-LD^16^) chains A and B (red) superimposed onto PDB entry 4jra (BoNT/A1-H_C_ – human SV2C-LD^12^) chains A and D (green), 496 residues aligned, core rmsd = 0.7036 Å; PDB entry 5jlv (BoNT/A1-H_C_ – human glycosylated SV2C-LD^16^) chains A and C (blue) superimposed onto PDB entry 4jra chains A and D, 495 residues aligned, core rmsd = 0.8930 Å; BoNT/A2-H_C_ - human SV2C-LD chains A and B (yellow) superimposed onto PDB entry 4jra chains A and D, 495 residues aligned, core rmsd = 0.9579 Å. The arrow indicates where the BoNT/A-H_C_ loop extends around the corner of the SV2C-LD β-helix. (**b**) The shallow binding cleft at the BoNT/A2 interaction site. BoNT/A2-H_C_ is depicted as a cartoon (green) and as a surface representation (light grey). SV2C-LD is shown as a cartoon (blue). Residue numbers are indicated for Thr 1145, Thr 1146 and Ser 1294. The residues are shown as sticks (N = blue, O = red).

In order to understand how BoNT/A2 can compensate for the missing interactions seen in the BoNT/A1-H_C_-SV2C-LD complex structure, we determined the crystal structure of SV2C-LD bound to BoNT/A2-H_C_ (Table 1). As in the previous BoNT/A1-H_C_ – SV2C-LD structures, the quadrilateral β-helix structure formed by SV2C-LD binds to a two-stranded, antiparallel β-sheet on the convex side of BoNT/A2H_C_. In the extended β-sheet, the interaction is dominated by backbone-to-backbone interactions. The overall C_α_-backbone architecture of the four known BoNT/A-H_C_ – SV2C-LD complexes is nearly identical (Fig. 2). BoNT/A2-H_C_ and SV2C-LD are both rigid and well defined in most regions. Notable exceptions where conformations differ between BoNT/A crystal structures, are the structurally variable, partially flexible loops between residues 1225 and 1235, 1262 and 1280 (both may be influenced by the presence or absence of a disulfide bond between Cys 1235 and Cys 1280) and 1166 and 1173. At the N-terminal side of H_C_, the rigid core starts roughly at residue 879 and the C-terminal side starts becoming structurally variable after residue 1294. The SV2C-LD β-helix core structure starts roughly at Lys 477 and ends at His 564, right at the end of the BoNT-interacting region. Upstream of residue 477, the conformation of the polypeptide differs greatly between crystal structures. In the BoNT/A2-H_C_ – human SV2C-LD structure, the polypeptide chain in this region forms a long extension that makes a crystal contact with a symmetry-related BoNT molecule.

### Comparison of the BoNT/A-H_C_ – SV2C-LD binding interfaces

**Figure 3:**
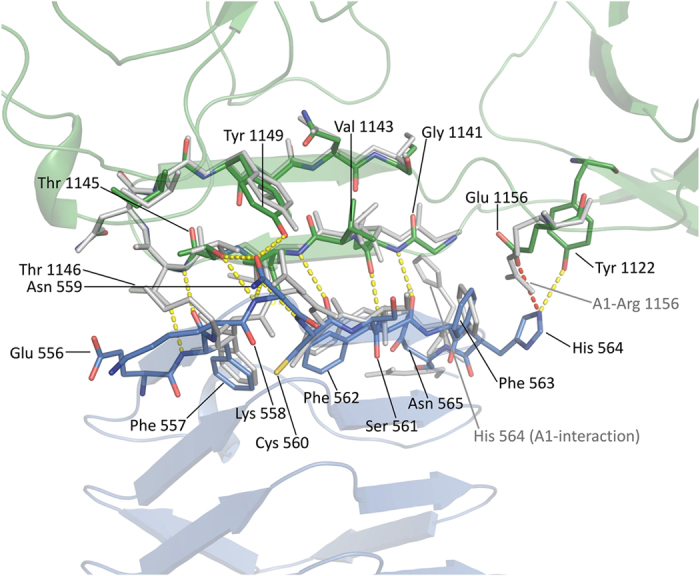
Comparison of the toxin-receptor interface of BoNT/A1-H_c_ and BoNT/A2-H_c_. The BoNT/A2-H_C_ (green) – human SV2C-LD (blue) interaction site is shown as sticks. Dark blue denotes nitrogen, red denotes oxygen and yellow denotes sulphur. Hydrogen bonds are shown as yellow dashed lines, the salt bridge is shown as a red dashed line. The interface of the superimposed BoNT/A1-H_C_ – human SV2C-LD complex (PDB entry 4jra^12^) is shown as sticks (grey). In the A1 structure, the face of the Phe 563 electron π-system stacks with the positively charged side chains of Arg 1156. In the A2 structure, residue 1156 is a negatively charged Glu and Phe 563 adopts a different conformation, facing Glu 1156 with the rim of the π-system.

All previously published structures of the complex contained BoNT/A1, while the structure we describe here is the first one containing a different subtype of the toxin, A2. There are a few side-chain interactions that are common to all known BoNT/A-H_C_ – SV2C-LD complex structures (Fig. 3). The OH group of BoNT/A Tyr 1149 and the OD1 of SV2C-LD Asn 559 form a hydrogen bond and Thr 1145 OG1 of the toxin forms hydrogen bonds to N and OD1 of Asn 559 of the receptor. While Asn 559 plays a key role in the interaction through its glycosylation^16^, this residue hence also contributes polypeptide side-chain interactions. Considering that an Asn 559 to Ala mutation did not significantly affect toxin-receptor binding in pulldown and fluorescence anisotropy experiments^12^, the hydrogen bonds involving Asn 559 do not appear to play a key role in the interaction. The next amino acid in the BoNT/A sequence, Thr 1146, forms hydrogen bonds through OG1 and N to Phe 557 N and O, respectively. This extends the β-strand-to-β-strand interaction towards the loop connecting the two receptor-interacting β-strands in BoNT/A and towards the edge of the β-helix in SV2C-LD. Simultaneous mutation of BoNT/A1 residues Thr 1145A and Thr 1146A resulted in significantly lower binding to SV2C-LD^12^, which was confirmed by Yao *et al*.^16^. These residues are conserved between subtypes (Fig. 1)^6^,^11^,^16^.

The structure of the BoNT/A2-H_C_ complex provides an explanation why human SV2C-LD can still bind strongly to toxin subtype A2 despite the pronounced sequence differences in key residues to the A1 subtype. Specific interactions compensate for the lacking cation-π stacking (Fig. 3). SV2C-LD His 564 NE2 forms a salt bridge with BoNT/A2-H_C_ Glu 1156 OE2 and a hydrogen bond with BoNT/A2-H_C_ Tyr 1122 OH. Furthermore, Glu 1156 of the toxin engages in an anion- π stacking interaction to Phe 563 of SV2C-LD. It therefore appears that the toxin has evolved to have a high plasticity for receptor recognition, tolerating a variety of side-chain interactions at the interface.

Recent data support the notion of high side-chain interaction plasticity. The structure of the BoNT/A1-H_C_ - SV2C-LD (rat)^16^ complex showed that this interaction also is not dependent on the cation-π stacking, since rat SV2C contains a leucine instead of a phenylalanine at position 563.

Arg 1294 of BoNT/A1-H_C_, which was shown to be important for the BoNT/A1-H_C_ – SV2C-LD (human) interaction^12^, is another residue that differs significantly between the structures. In the initial structure^12^, this residue was poorly defined in one of the two complexes in the asymmetric unit (in chain B) and appeared to contribute to the overall positive charge of the binding site of the BoNT domain. In the case of BoNT/A1-H_C_ – SV2C (rat) structure, Arg 1294 is well defined and forms hydrogen bonds to Ser 519, Cys 520, Thr 521 and Asp 539^16^, which may stabilize the interaction. In BoNT/A2-H_C_, residue 1294 is a serine, which does not engage in polar contacts with the receptor. These data suggest that the charge of residue 1294 is less important for SV2C binding than originally anticipated.

### Motif extensions contribute to binding specificity

Yao *et al*.^16^ suggested that the variability of side-chains at the BoNT/A - SV2C interface is tolerated because N-glycosylation of SV2C at Asn 559, which contributes to BoNT/A binding, is highly conserved. While the data of Yao *et al*.^16^ indicate that SV2C Asn 559 glycosylation slightly enhances BoNT/A1-H_C_ binding by a factor of four, the currently available structural information strongly suggests that the proteinaceous interactions are sufficient to mediate binding specificity: Amongst the four known crystal structures of the toxin-receptor complex, only one contains glycosylated SV2C-LD, yet all of them show a nearly identical binding mode (Fig. 2). A plausible explanation how specificity may be achieved without an extensive side-chain interaction network lies in the overall fit between the shape of the toxin and the shape of the receptor at the interaction site. SV2C-LD adopts a quadrilateral β-helix, a structure that is rare in human proteins. The open β-strand of BoNT/A-H_C_ does not simply bind to one open strand of the β-helix, but forms a motif extension beyond the corner to the next strand (Figs 2 and 3). Thr 1145 and Thr 1146 are located in the loop connecting the two BoNT/A-H_C_ β-strands at the binding interface. The hydrogen bonds between BoNT/A-H_C_ Thr 1145 OG1 and SV2C-LD Asn 559 N and OD1, between BoNT/A-H_C_ Thr 1146 OG1 and SV2C-LD Phe 557 N as well as between BoNT/A-H_C_ Thr 1146 N and SV2C-LD Phe 557 O extend the interaction around the β-helix corner, towards the SV2C-LD exposed open β-strand of the subsequent SV2C-LD β-helix side.

BoNT/A-H_C_ forms a shallow cleft that fits the β-helix corner (Fig. 2). While one side of the binding cleft of the toxin is defined by the open β-strand, the C-terminal tail of BoNT/A-H_C_ sterically delimits the positioning of SV2C-LD at the face of its main interacting β-strand and loop. Although residue 1294 is not conserved between BoNT/A subtypes, and despite the different side-chain conformations that have been observed for this amino acid in different X-ray structures, the C-terminal tail of BoNT/A-H_C_ in all published structures forms a surface protrusion that sterically delimits one side of the binding site cleft of the toxin.

### Crystal structures are consistent with recent cellular data

For BoNT/A, eight different subtypes have been identified, some of which differ in their intoxication properties^7^,^8^. For example, Pier and colleagues^17^ reported that BoNT/A2 is more potent in neuronal cells than BoNT/A1, data that are consistent with recent *in vivo* studies^18^. They found that the mechanism explaining the difference in toxicity is a faster and more efficient cell entry of BoNT/A2 that is independent of ganglioside binding. In support of these findings, BoNT/A2 showed increased co-localization with synaptic vesicles compared to BoNT/A1. In agreement with this observation, Kroken and colleagues^13^ also observed more efficient entry of BoNT/A2 relative to BoNT/A1, although the authors found similar initial rates of cell entry for both subtypes. Kroken *et al*.^13^ showed sequential entry of both BoNT/A1 and A2 with an initial step requiring a ganglioside association step and a subsequent SV2-dependent step. Interestingly, BoNT/A2 showed increased receptor occupancy compared to BoNT/A1, demonstrating that both subypes use the same receptors for neuronal entry, but differ with regard to their affinity for the receptors. Experiments with a BoNT/A1 mutant in which a four-residue loop (amino acids 1271–1274) adjacent to the ganglioside-binding site was replaced by the one of A2 demonstrated intermediate neuron entry properties compared to the two subtypes. This finding confirmed the role differential ganglioside affinity in toxin entry of BoNT/A1 and A2 subtypes.

Our results are consistent with the ganglioside-binding experiments performed by Kroken and colleagues^13^ because currently available structural information argues against major differences in SV2C binding of the two subtypes. A comparison of toxin-receptor complex structures revealed an identical interaction mode of both subtypes to the protein moiety of SV2C and a conservation of the residues involved in SV2C- carbohydrate binding^11^,^12^,^16^ (Fig. 2).

### Conclusion/Outlook

It took almost a decade from the identification of SV2 as the protein receptor of BoNT/A^19^,^20^ until a first crystal structure of a toxin-receptor complex^12^ was elucidated. Three follow-up complex structures have in the meantime been solved (ref. 16 and the structure described here). The currently available structural information indicates a common binding mode for different BoNT/A subtypes and for SV2C luminal domains from different species. Notably, N-glycosylation only results in a ~4-fold lowering of the K_d_ of complex dissociation^16^ and there is no significant difference in the overall binding mode between complexes comprising glycosylated or non-glycosylated SV2C-LD. These findings strongly suggest that the specificity of recognition and binding by the toxin is dominated by the proteinaceous part of the receptor. This conclusion will be tested when further complex structures of additional BoNT/A subtypes and SV2C-LDs from additional species will become available in the near future.

## Materials and Methods

### Cloning

Cloning of SV2C-LD and BoNT/A1-H_C_ has previously been described^12^. The cDNAs encoding human SV2C (Swiss-Prot Q496J9) amino acids 456–574 and BoNT/A (Swiss-Prot P10845) amino acids 871–1296 were amplified by PCR using DNA templates that were codon-optimized for expression in Escherichia coli (GENEWIZ) and cloned into a modified pET-based vector containing an N-terminal 6xHis-tag by co-transformation cloning^21^,^22^.

A codon optimized DNA fragment (GENEWIZ) of BoNT/A2-H_C_ was amplified by PCR using primers 5-CCGCGTGGATCCAAGAACATTGTGAACACCAGTATTCTGAGCATTG-3′ and 5′-ATCTTAGAATTCTTATTACAGGCTGCTTTCACCCCAGCCGTC-3′ and cloned into the BamHI and EcoRI sites of the vector pHisTrx^23^.

### Protein expression and purification

The proteins were expressed in BL21(DE3) *E. coli* cells at 20 °C overnight. The cell pellets were resuspended in 50 mM Tris pH 7.5, 500 mM NaCl, 20 mM Imidazole, 10 mM β-mercaptoethanol supplemented with Roche EDTA-free protease inhibitor cocktail. Lysis was performed by ultrasonication. BoNT/A1-H_C_, BoNT/A2-H_C_ and SV2C-LD were first separately purified by Ni-NTA IMAC (Wash buffer: 50 mM Tris pH 7.5, 500 mM NaCl, 60 mM Imidazole, 10 mM β-mercaptoethanol, eluted with an imidazole step gradient) and size-exclusion chromatography (HiLoad 16/60 Superdex 200 column; Buffer: 50 mM Tris pH 7.5, 150 mM NaCl, 20 mM Imidazol, 10 mM β-mercaptoethanol). The 6xHis-TrxA-BoNT/A2-H_C_ construct was cleaved with thrombin, and the 6xHis-tagged TrxA fusion protein was removed in a second (reverse) IMAC step. For crystallization, the two protein domains were mixed (1.5 × molar excess of SV2C-LD over BoNT/A2-H_C_), incubated on ice for 1 hour, concentrated using a 10 kDa cutoff centrifugal filter device (Amicon), and run over a gel filtration column again (HiLoad 16/60 Superdex 200). The eluted complex was concentrated to 14 mg/ml and used for crystallization trials.

### Crystallization and structure elucidation

Crystals were grown in hanging drops at 12 °C, using the streak seeding method (from crystals obtained in condition D4 of the Qiagen PEGs Suite screen, 100 mM Hepes, pH 7.5, 25% PEG 8000) for crystal optimization. The reservoir solution consisted of 20% PEG 8000, 65 mM Hepes, pH 7.5. The cryoprotectant consisted of 20% PEG 8000, 65 mM Hepes, pH 7.5, 15% glycerol.

A complete dataset to 2.3 Å resolution was collected from a single crystal at 100 K at the X06DA beamline of the Swiss Light Source synchrotron in Villigen, Switzerland, using a wavelength of 1.000 Å. Data processing was carried out using XDS^24^. The high-resolution limit was chosen according to the cc1/2^25^. The crystal belonged to space group P2_1_2_1_2_1_. The structure of the BoNT/A2-H_C_ – SV2C-LD complex was solved by molecular replacement (Phaser^26^), using a Phyre2^27^ model (based on pdb entry 2vu9^28^) of BoNT/A2-H_C_, and residues 476–565 of the SV2C-LD from the BoNT/A1-H_C_ – SV2C-LD structure^12^ (pdb entry 4jra), as the search models. One complex could be positioned in the P2_1_2_1_2_1_ asymmetric unit by using Phaser^26^. The atomic model was refined with phenix.refine^29^. Coot^30^ was used for manual model building. The final refined structure exhibits good geometry and stereochemistry, as validated with MolProbity^31^.

Secondary-structure matching superimpositions^32^ were carried out with SSM superpose as implemented in Coot^30^. Structure figures were prepared using PyMOL^33^.

The structure was deposited in the PDB under the accession code: 5MOY.

### Pulldown assays

Purified 6xHis-TrxA-tagged BoNT/A2-H_C_ or 6xHis-BoNT/A1-H_C_ and untagged SV2C-LD were separately centrifuged at 16,000 g for 15 minutes at 4 °C. The proteins (final concentrations: 5 μM) were then combined and incubated overnight at 4 °C in pulldown buffer (20 mM Tris, pH 7.8, 150 mM NaCl and 5 mM β-mercaptoethanol). After incubation the samples were again centrifuged. The supernatants were incubated with ~50 μl Ni-Sepharose 6 Fast Flow resin (GE Healthcare) for 60 minutes. Next, the resin was washed four times with 2 ml ice-cold pulldown buffer and the proteins were eluted in 75 μl pulldown buffer supplemented with 400 mM imidazole, followed by analysis on SDS-PAGE.

## Additional Information

**How to cite this article:** Benoit, R. M. *et al*. Crystal structure of the BoNT/A2 receptor-binding domain in complex with the luminal domain of its neuronal receptor SV2C. *Sci. Rep.* **7**, 43588; doi: 10.1038/srep43588 (2017).

**Publisher's note:** Springer Nature remains neutral with regard to jurisdictional claims in published maps and institutional affiliations.

## Figures and Tables

**Table 1:** Data collection and refinement statistics.

Data collection	
Space group	P 2_1_2_1_2_1_
Cell dimensions	
*a, b, c* (Å)	47.738, 91.573, 147.663
*α, β, γ* (°)	90.0, 90.0, 90.0
Resolution (Å)	50–2.3 (2.44–2.30)
R_meas_	11.8 (177.2)
CC_1/2_	99.8 (54.8)
I/SigmaI	12.56 (1.07)
Completeness (%)	99.9 (100.0)
Redundancy	6.5 (6.8)
Refinement	
Resolution (Å)	45.8–2.3
No. reflections	29489
R_work_/R_free_	0.195/0.251
No. atoms	
Protein	4801
Water	76
B-factors	
Protein	59.2
Water	46.4
R.m.s. deviations	
Bond lengths (Å)	0.010
Bond angles (°)	1.017

The highest resolution shell is shown in parentheses.
